# Publication Rate and Characteristics of Lung Cancer Clinical Trials

**DOI:** 10.1001/jamanetworkopen.2019.14531

**Published:** 2019-11-06

**Authors:** Ghassan Al-Shbool, Hira Latif, Saira Farid, Shuqi Wang, Jaeil Ahn, Giuseppe Giaccone, Chul Kim

**Affiliations:** 1 MedStar Washington Hospital Center, Washington, DC; 2 Department of Biostatistics, Bioinformatics and Biomathematics, Georgetown University, Washington, DC; 3 Lombardi Comprehensive Cancer Center, Georgetown University, Washington, DC

## Abstract

This cross-sectional study estimates the publication rate of clinical trials related to lung cancer and examines the characteristics of published vs unpublished trials.

## Introduction

Clinical trials play a crucial role in shaping the practice of medicine. Nonreporting of clinical trial results affects the validity of the literature and can negatively affect patient care. Lung cancer is the leading cause of cancer-related mortality in the United States and worldwide.^1^ Studies assessing reporting of clinical trial results in thoracic oncology are lacking. We sought to investigate the publication rate among registered lung cancer clinical trials.

## Methods

For this cross-sectional study, ClinicalTrials.gov was searched on August 8, 2018, to identify registered lung cancer clinical trials with a study completion date between January 1, 2000, and August 7, 2016. Interventional phase 2 or 3 trials involving patients aged 18 years or older with a recruitment status of completed or terminated were included. Using the National Clinical Trial identifier, trial title, and keywords, 3 databases were searched. PubMed and Google Scholar were searched by 2 of us independently (G.A.S. and S.F.). Another of us (C.K.) searched EMBASE for the trials found unpublished after the initial literature search. Subsequently, email queries were sent to trial investigators of unpublished completed trials. Trial results were categorized as (1) publication in a peer-reviewed journal and (2) no peer-reviewed publication (including conference proceedings and reporting of results on ClinicalTrials.gov). Logistic regression analysis was performed to assess the characteristics associated with nonpublication. Statistical analysis was performed using R statistical software version 3.4 (R Project for Statistical Computing). The date of analysis was July 15, 2019. The study follows the Strengthening the Reporting of Observational Studies in Epidemiology (STROBE) reporting guideline. In accordance with the policies of the Human Subject Protection Program at Georgetown University Medical Center, this study was not submitted for ethical review and did not require informed consent. 

## Results

A total of 1294 clinical trials were included (Figure). In all, 1038 (80.2%) were completed and 256 (19.8%) were terminated. The median (interquartile range) follow-up time after study completion or termination was 116 (81-147) months. Characteristics of completed trials are summarized in the Table. Among the 1038 completed trials, our search of the databases found that 702 (67.6%) were published and 336 (32.4%) were unpublished. Among the 336 unpublished clinical trials, we identified the contact information of trial investigators for 183 clinical trials (54.5%). We received 102 responses (55.7%); 51 reported peer-reviewed publication of their trials and 51 confirmed the nonpublication status. Overall, among the completed trials, we found that 753 trials (72.5%) were published and 285 trials (27.5%) were unpublished. Industry-sponsored trials were less likely to be published compared with those sponsored by the National Institutes of Health (odds ratio [OR], 2.27; 95% CI, 1.31-4.00) and academic institutions (OR, 1.55; 95% CI, 1.15-2.10) (Table). Multicenter studies were more likely to be published (OR, 2.78; 95% CI, 2.08-3.73) than single-center studies. Greater patient enrollment was associated with publication (>500 participants: OR, 3.43; 95% CI, 1.83-6.64; and 100-500 participants: OR, 2.87; 95% CI, 2.08-3.73) compared with studies with fewer than 100 participants. Randomization status and trial phase were not associated with publication. Among the 256 terminated clinical trials, 72 (28.1%) were published in peer-reviewed journals, while 184 (71.9%) remained unpublished. Trials were terminated for various reasons: low enrollment, 119 (46.5%); lack of efficacy, 30 (11.7%); toxic effects, 22 (8.6%); miscellaneous (eg, principal investigator left the institution), 38 (14.8%); and unknown, 47 (18.4%).

**Figure.**
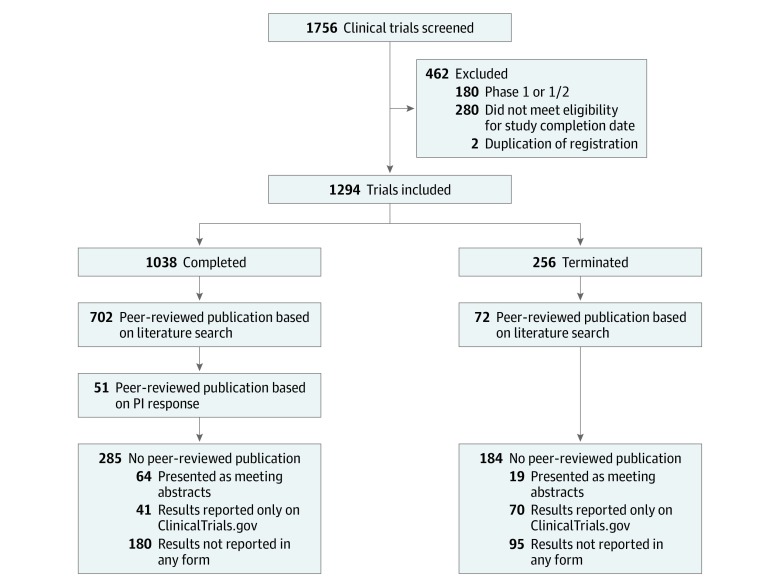
Flowchart of Included Clinical Trials PI indicates principal investigator.

**Table.** Characteristics of Completed Trials and Their Association With Peer-Reviewed Publication Status

Characteristic	No. (%)	Estimated OR (95% CI)
No. of completed clinical trials	1038	
Phase		
2	799 (76.9)	1 [Reference]
3	239 (23.0)	0.66 (0.43-1.00)
Patient enrollment, No.		
<100	590 (56.8)	1 [Reference]
100-500	332 (32.0)	2.87 (2.08-3.73)
>500	100 (9.6)	3.43 (1.83-6.64)
Not reported	16 (1.5)	
Primary sponsor		
Industry	415 (40.0)	1 [Reference]
National Institutes of Health	77 (7.4)	2.27 (1.31-4.00)
Academic institutions	546 (52.6)	1.55 (1.15-2.10)
Randomization		
Nonrandomized	522 (50.3)	1 [Reference]
Randomized	516 (49.7)	1.27 (0.92-1.76)
Duration of the trial, y		
<2	228 (22.0)	1 [Reference]
2-5	578 (55.7)	1.66 (1.24-2.24)
>5	215 (20.7)	2.08 (1.40-3.10)
Not reported	17 (1.6)	
Location		
Single center	292 (28.1)	1 [Reference]
Multicenter	730 (70.3)	2.78 (2.08-3.73)
Not reported	16 (1.5)	

Abbreviation: OR, odds ratio.

## Discussion

Among lung cancer clinical trials registered in ClinicalTrials.gov and completed prior to 2016, 1 in 4 trials remained unpublished, raising the concern of publication bias in the field of thoracic oncology. The rate of nonpublication was high among terminated trials and more than a third did not report their study results in any form. Our findings are similar to previously reported rates of nonpublication among trials in oncology and other disciplines.^2,3,4,5^

There are, however, several limitations to our analysis. First, we relied on information available in ClinicalTrials.gov, which is provided by the trial sponsor or investigator, and the accuracy of the data could not be verified. Second, we included trials completed by August 2016, and it is possible that some trials might have been published after our analysis was completed. Third, our search for publications, albeit comprehensive, might have missed some studies.

Timely publication of clinical trial findings is instrumental in informing oncology practice and future research. Also, the ethical standards set out in the Declaration of Helsinki obligate researchers to report the results of their work regardless of the results. Several organizations have made attempts to decrease the rate of nonpublication. The AllTrials campaign, an international initiative, calls for registration and reporting of all trials.^6^ There are oncology journals encouraging the reporting of negative results from clinical trials. Recently, medRxiv, a preprint server for clinical research, was launched and hosts manuscripts, including those of clinical trials. Clinical trials rely on the willingness of patients to risk exposure to unproven interventions. Reporting trial results is our obligation and responsibility to trial participants.
